# Examination of internal metabolome and VOCs profile of brewery yeast and their mutants producing beer with improved aroma

**DOI:** 10.1038/s41598-024-64899-4

**Published:** 2024-06-25

**Authors:** Sławomir Jan Jabłoński, Karolina Anna Mielko-Niziałek, Przemysław Leszczyński, Alan Gasiński, Joanna Kawa-Rygielska, Piotr Młynarz, Marcin Łukaszewicz

**Affiliations:** 1 Department of Biotransformation, Faculty of Biotechnology, University of Wrocław, Wrocław, Poland; 2 Department of Biochemistry, Molecular Biology and Biotechnology, Faculty of Chemistry, Wrocław University of Science and Technology, Wrocław, Poland; 3 Department of Fermentation and Cereals Technology, Faculty of Biotechnology and Food Science, Wrocław University of Environmental and Life Sciences, Wrocław, Poland

**Keywords:** Volatile organic compounds, Yeast, Metabolomics, Biochemistry, Biotechnology

## Abstract

Volatile organic compounds (VOCs) are metabolites pivotal in determining the aroma of various products. A well-known VOC producer of industrial importance is *Saccharomyces cerevisiae*, partially responsible for flavor of beers and wines. We identified VOCs in beers produced by yeast strains characterized by improved aroma obtained in UV-induced mutagenesis. We observed significant increase in concentration of compounds in strains: 1214uv16 (2-phenylethyl acetate, 2- phenylethanol), 1214uv31 (2-ethyl henxan-1-ol), 1214uv33 (ethyl decanoate, caryophyllene). We observed decrease in production of 2-phenyethyl acetate in strain 1214uv33. Analysis of intracellular metabolites based on ^1^H NMR revealed that intracellular phenylalanine concentration was not changed in strains producing more phenylalanine related VOCs (1214uv16 and 1214uv33), so regulation of this pathway seems to be more sophisticated than is currently assumed. Metabolome analysis surprisingly showed the presence of 3-hydroxyisobutyrate, a product of valine degradation, which is considered to be absent in *S. cerevisiae*. Our results show that our knowledge of yeast metabolism including VOC production has gaps regarding synthesis pathways for individual metabolites and regulation mechanisms. Detailed analysis of 1214uv16 and 1214uv33 may enhance our knowledge of the regulatory mechanisms of VOC synthesis in yeast, and analysis of strain 1214uv31 may reveal the pathway of 2-ethyl henxan-1-ol biosynthesis.

## Introduction

Volatile organic compounds (VOCs) are diverse metabolites produced by various organisms, playing a pivotal role in ecological interactions and industrial applications^1,2^. VOC’s produced by *Saccharomyces cerevisiae* play an important role in flavor development of many fermented beverages, such as wine, beer, cider, as well as other alcoholic beverages produced from peculiar fruits, honey or tea. It is hypothesized that yeasts have evolved VOC production pathways to attract insects, leveraging them as vectors for environmental spread^3^.

In the context of alcoholic beverages like ales and wines, *S. cerevisiae’s* VOCs are critical for flavor development^4^. The impact of these compounds on the overall aroma is influenced by their concentration and respective odor thresholds (see Table 1). For instance, ethyl butanoate, with a low odor threshold, affects beverage aroma at lower concentrations, whereas compounds like 2-phenylethanol require higher concentrations due to their higher odor thresholds. These characteristics make certain VOCs also valuable to other industries, including chemicals, cosmetics, and fuel.

**Table 1:** Odor threshold and aroma impression for selected VOCs.

Compound name	CAS number	Class	Odor thresholds [mg/L]	Aroma impression	Reference
Ethyl butanoate	105-54-4	Ethyl ester	0.4–0.45	Pineapple	^11,12^
Ethyl hexanoate	123-66-0	Ethyl ester	0.2	Fruity, apple Peel	^13^
Ethyl octanoate	106-32-1	Ethyl ester	0.9	Fruity, flowers, apricot	^13^
Ethyl decanoate	110-38-3	Ethyl ester	1.5	Fruity, cognac	^13^
Ethyl dodecanoate	106-33-2	Ethyl ester	2	Berry, grape, nut, rum, spice	^14^
2-methylpropyl acetate (isobutyl acetate)	110-19-0	Acetate ester	0.7–1.0	Sweet, apple, tropical, banana	^12,15^
3-methylbutan-1-ol acetate (isoamyl acetate)	123-92-2	Acetate ester	1.6	Banana, pear	^16^
2-phenylethyl acetate	103-45-7	Acetate ester	0.2–3.8	Fruit, rose, honey	^13^
2-phenylethanol	60-12-8	Alcohol	7.5–125	Rose	^13,17^
2-ethyl hexan-1-ol	104-76-7	Alcohol	0.075	Earthy, floral, citrus	^18^
1-decanol	112-30-1	Alcohol	0.006	Fatty, sweet, orange	^19^
Linalool	78-70-6	Terpenoid	0.006	Intense floral, lavender	^20^
Caryophyllene	87-44-5	Terpenoid	0.15	Musky, spicy, earthy, sweet	^20^
Nerolidol	142-50-7	Terpenoid	15	Herbal, floral, woody	^21^
Camphor	76-22-2	Terpenoid	No data available	Specific	
Isopropyl myristate	110-27-0	Ester	None	None	

VOCs in yeast span several chemical groups, including volatile alcohols, esters, and terpenoids, originating from basic metabolic processes like glycolysis, amino acid metabolism, and lipid metabolism^5^. Enhancing VOC production in yeast strains might not only augment the attractiveness of beverages but also reduce reliance on additional flavoring agents, offering cost benefits and alternative sources for the cosmetic industry.

Despite the majority of VOC synthesis pathways being known, developing high-producing strains with simple over production of individual proteins has achieved only moderate success^6^. This could be due to unrecognized regulatory mechanisms in VOC synthesis pathways. Additionally, societal acceptance of genetically modified organisms, including yeast, varies globally, making random mutagenesis an appealing alternative for strain development.

Analysis of the strain library following random mutagenesis could unveil new insights into *S. cerevisiae*. Although bakery yeast is a pivotal model organism in scientific research, with its genome being the first eukaryote to be sequenced, the functions of approximately 10% of its identified genes are still a mystery. Moreover, the metabolic pathways for certain yeast-produced metabolites have not been fully delineated. An illustrative case is 2-ethylhexanol, a compound found in some wines and beers, whose biosynthesis pathway in yeast remains elusive^7^. Research on the growth of *Carnobacterium maltaromaticum* on meat revealed that formation of this compound is specific to this bacteria among other species isolated from meat^8^.

Elucidating unknowns may be resolved with inverted metabolic engineering^9^. This concept involves a detailed comparison of two (or more) closely related yeast strains, of which at least one possesses the required trait (high production of the desired compound). In an ideal situation, compared data would involve genomic data, gene expression data, and metabolomic data. If only partial data are available, it may still be possible to identify the cause of the beneficial change. Inverted metabolic engineering was successfully used in research on the production of erythritol by *Yarrovia lipolitica* yeast^10^.

In this study, we analyze a collection of yeast strains generated through UV light-induced random mutagenesis. These strains were initially selected based on the desirable aromas they produced during wort fermentation. We identified and quantified the main compounds contributing to this aroma, discovering that individual strains significantly enhanced the production of certain VOCs. Notably, the strain PP08uv07 exhibited a marked increase in the concentration of all detected terpenoids, while 1214uv31 produced tenfold more 2-ethyl hexan-1-ol. To explore potential alterations in the metabolic pathways that could account for the changed VOC production, we measured the relative concentration of intracellular metabolites employing the ^1^H NMR technique.

## Results and discussion

In our research, we analyzed the concentrations of selected VOCs in beers produced with specific yeast strains, PP08 and 1214, and their mutants generated through random mutagenesis using UV light. The list of analyzed VOCs is presented in Table 1. The set of analyzed VOCs included 9 esters (5 ethyl esters and 3 acetate esters), 3 alcohols, and 4 terpenoids.

**Figure 1:**
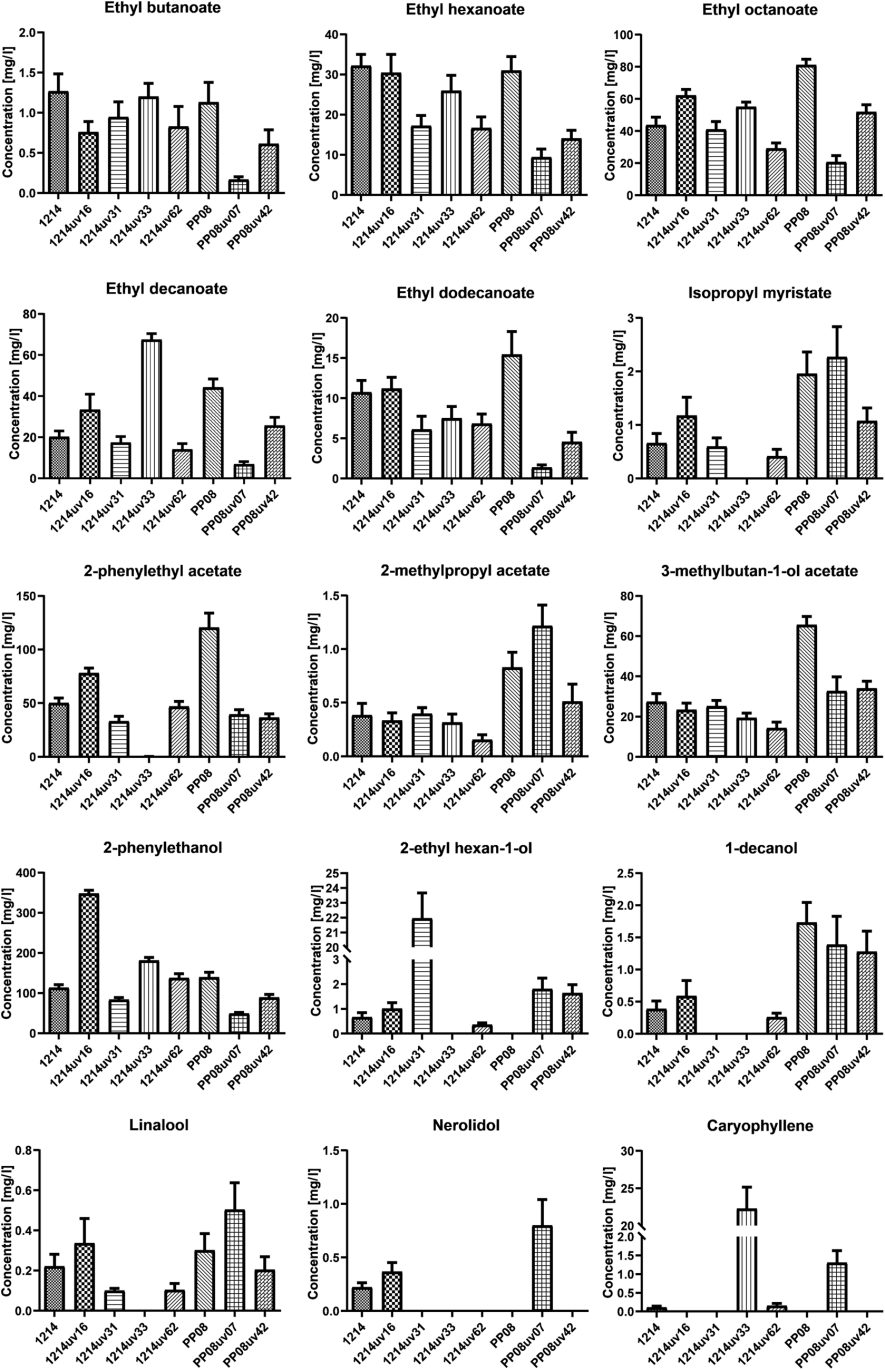
Concentration of individual VOCs in beer fermented by different yeast strains.

The concentration of individual VOCs identified in the beers is depicted in Fig. 1. Detailed statistical analysis of these results is available in Table S2 of the supplementary materials. The samples analyzed demonstrated significant variability in VOC concentrations, with p-values below 1.5% across all identified VOCs. Thus, at least partial differences in beer aroma may be explained by altered concentrations of individual compounds in the analyzed set. When comparing the observed concentrations of individual VOCs with available odor thresholds (Table 1), it appears that the majority of the detected aroma compounds likely influence beer aroma. However, the concentrations of 2-phenylethanol and 2-methylpropyl acetate are close to their odor thresholds, suggesting their contribution to the final aroma might be minimal. Nerolidol most likely did not affect beer aroma since its concentration was below the odor  threshold in all analyzed samples. Caryophyllene concentration significantly exceeded the odor threshold in the case of strains 1214uv33 and PP08uv07.

The concentration of identified acetate esters (except isobutyl acetate) was around 10 times higher when compared with values measured in beers and wines produced with other yeast strains^22,23^. Acetate esters, such as isoamyl acetate, phenylethyl acetate, and isobutyl acetate, were thought to be synthesized from amino acid precursors in the wort (or must) via the Ehrlich pathway. This pathway involves several steps: the removal of an amino group from an amino acid, decarboxylation of the resulting keto acid, reduction to the corresponding alcohol, and finally, the formation of an acetate ester^24^. Simplified interpretation of this pathway assumes that the produced mixture of amino acid-related VOCs should reflect the availability of corresponding amino acids in the wort. The final concentration of acetate esters would also be limited by the initial amount of amino acids in the culture medium. Adding amino acids should enhance the formation of corresponding higher alcohols and their esters. This is exemplified with leucine; its addition led to a two-fold increase in isoamyl alcohol and isoamyl acetate concentrations^25^. Moreover, when we compare the average concentration of leucine in wort (around 150 mg/L)^26^ with the level of isoamyl acetate in the final product (30 mg/L), we may assume that a substantial part of leucine was converted to the corresponding ester.

However, alfa-keto acids may be derived from the amino acid synthesis pathway, thus amino acid-related VOCs may be also produced from sugar. It was confirmed in experiments including fermentation of ^13^C labeled glucose in wine making^23^, where authors show that around 90% of amino acid-related VOCs were derived from glucose. Our findings indicate that the production of 2-phenylethanol and 2-phenylethyl acetate by the analyzed strains is dependent rather on sugar than on phenylalanine from the wort. The summed concentrations of phenylalanine-related VOCs are at least close to the concentration of free phenylalanine in fresh wort (approximately 100 mg/L)^26^. Additionally, the total concentration of these VOCs in the case of strain 1214uv16 (around 430 mg/L) and concentration of 2-phenylethanol is significantly higher in comparison to other strains (*p* < 0.01%). Increased production of phenylpyruvate derived from Shikimate pathway, caused by UV induced mutations, may be directed to Ehrlich pathway and this would explain the observed results. In experiments with wine yeast strains increased production of phenylalanine related VOCs was the result of mutations in *aro4* gene^27^. Altered protein was insensitive to inhibition by phenylalanine and tyrosine. Similar situation may take place in the case of strain 1214uv16. If mutation in *aro4* is the only cause of increased production of 2-phenylethanol, one would expect that also concentration of phenylalanine and tyrosine should rise. However, in the case of strain 1214uv16, intracellular concentration of phenylalanine and tyrosine is not changed (see table S4 in supplementary materials), thus other alterations in regulation mechanisms may be present in this strain. 1214uv16 may be helpful in research on regulation of phenylalanine-related VOCs synthesis. Using strain 1214uv16 in beverage production could yield a strong rose aroma, irrespective of the wort’s phenylalanine concentration. This also opens up prospects for producing 2-phenylethanol in yeast for the cosmetic and chemical industries.

Another complexity within the Ehrlich pathway model is the presence of multiple enzymes with different substrate specificities catalyzing individual reactions. It is generally believed that yeast’s main acetate esters are synthesized by three alcohol acetyltransferases—Atf1p, Atf2p, and Eat1—with broad specificity^6^. However, our results challenge this assumption. In strain 1214uv33, we observed a very low concentration of 2-phenylethyl acetate (*p* < 2.5%) despite unchanged levels of other acetate esters. This suggests that contrary to previous understanding^6^, not all acetyltransferases may be capable of synthesizing 2-phenylethyl acetate. The near-complete inhibition of 2-phenylethyl acetate synthesis in this strain, even with available 2-phenylethanol, implies that a specific acetyltransferase, potentially unique to strain 1214, might be responsible for this synthesis and could have been altered in 1214uv33. Analyzing this strain may lead to the discovery of an acetyltransferase with narrow specificity, valuable for developing industrial strains specialized in producing specific esters.

In the analyzed set of strains mutations introduced by UV radiation had different effect on the concentration of ethyl esters. Strain PP08uv07 produced significantly lower concentration of all analyzed ethyl esters when compared with parental strain (*p* < 5%). Strain PP08uv42 significantly reduced production of ethyl hexanoate (*p* = 2.62%) and ethyl octanoate (*p* = 0.21%) in comparison to strain PP08. In case of 1214 mutants, most ethyl esters were not affected when compared to the parental strain. Decreased concentration was observed for ethyl hexanoate in 1214uv62 (*p* = 4.7%). Significantly increased concentration was observed in the case of ethyl decanoate in strain 1214uv33 (*p* < 0.5% in comparison to all strains derived from 1214). Close analysis of 1214uv33 may give better insight in understanding acyltransferases activity. The synthesis of ethyl esters in yeast is mainly carried out by acyltransferases Eeb1 and Eth1. These enzymes use acyl-CoA as a substrate for the transesterification reaction. Previous experiments using strains with overexpressed genes indicate that the concentration of ethyl esters is limited by the availability of carboxylic acids derived from the lipid synthesis pathway, rather than the enzyme concentration^28^. The addition of monocarboxylic acids to the medium significantly increased the concentration of related esters after fermentation. Decreased availability of substrates may explain the results observed for strain PP08uv07 where concentrations of all identified ethyl esters were significantly lower in comparison to the parental strain. The observed concentration ratio for carboxylic acids ethyl esters results from the specificity of acyl-CoA synthases (preferring longer substrates^29^) and acyltransferases (preferring substrates containing between 4 and 8 carbon atoms). Since acylo-CoA synthases prefer longer substrates, then availability of decano-CoA should not limit the production of ethyl decanoate. In case of strain 1214uv33, only concentration of ethyl decanoate was significantly altered, thus general availability of different acylo-CoA does not seem to be altered. A more likely cause may be a mutation leading to altered specificity of acyltransferases. Analysis of sequences coding for these proteins in strain 1214uv33 may give some clues about the organization of the substrate pocket in these enzymes.

Specific terpenoid VOCs may be produced by yeast as a byproduct of the sterol biosynthesis pathway^30^. Four terpenoid VOCs were identified in beers produced by analyzed yeast strains. Linalool was produced by all analyzed yeast strains except strain 1214uv33. Camphor was identified only in PP08uv07 with the concentration equal to 0924 ± 0442 mg/l. The highest concentration of caryophyllene was observed in 1214uv33, approximately an order of magnitude higher than the concentrations observed for other terpenoids. In beer produced with strain 1214uv33, the concentration of caryophyllene was high enough to influence the final product's aroma. Nerolidol was produce by strains 1214, 1214uv16 and PP08uv07, however concentration of this compound was far below its odor threshold.

Yeast as an alternative source of terpenoid VOCs may become attractive since the hops market is affected by climate changes. Majority of terpenoid VOCs in beer typically originate from hops. The quantity of terpenoids derived from hops and present in the final product is contingent on the beer production process. Hops-derived terpenoids may be lost due to evaporation, chemical transformation during boiling and fermentation, or absorption to solid particles such as yeast cells^31^. Increased production of terpenoid VOCs may be achieved by introducing genetic modifications to yeast strains^32^; however, it is not a solution for the beverage industry due to a lack of acceptance for GMOs in the market. Our results show that a similar effect may be obtained without molecular biology techniques. Moreover, a close analysis of strain 1214uv33 may help in the identification of molecular mechanisms underlying increased production of caryophyllene.

2-ethylhexanol was identified in beer produced by all strains except 1214uv33 and PP08. Only strain 1214uv31 produced significantly greater amount of this VOC (*p* < 0.01%). This metabolite was previously identified in different alcoholic beverages, including wine and beer^7^. It was also identified in the process of meat spoilage, and it was proven that the formation of this metabolite requires the activity of microorganisms (*Carnobacterium maltaromaticum*)^8^. However, the substrates and biochemical reactions leading to the formation of 2-ethylhexanol remain unidentified. The concentration of this metabolite achieved by strain 1214uv31 was an order of magnitude higher than that observed in other strains, making this strain a promising candidate for further investigation into the biosynthesis of 2-ethylhexan-1-ol.

**Figure 2:**
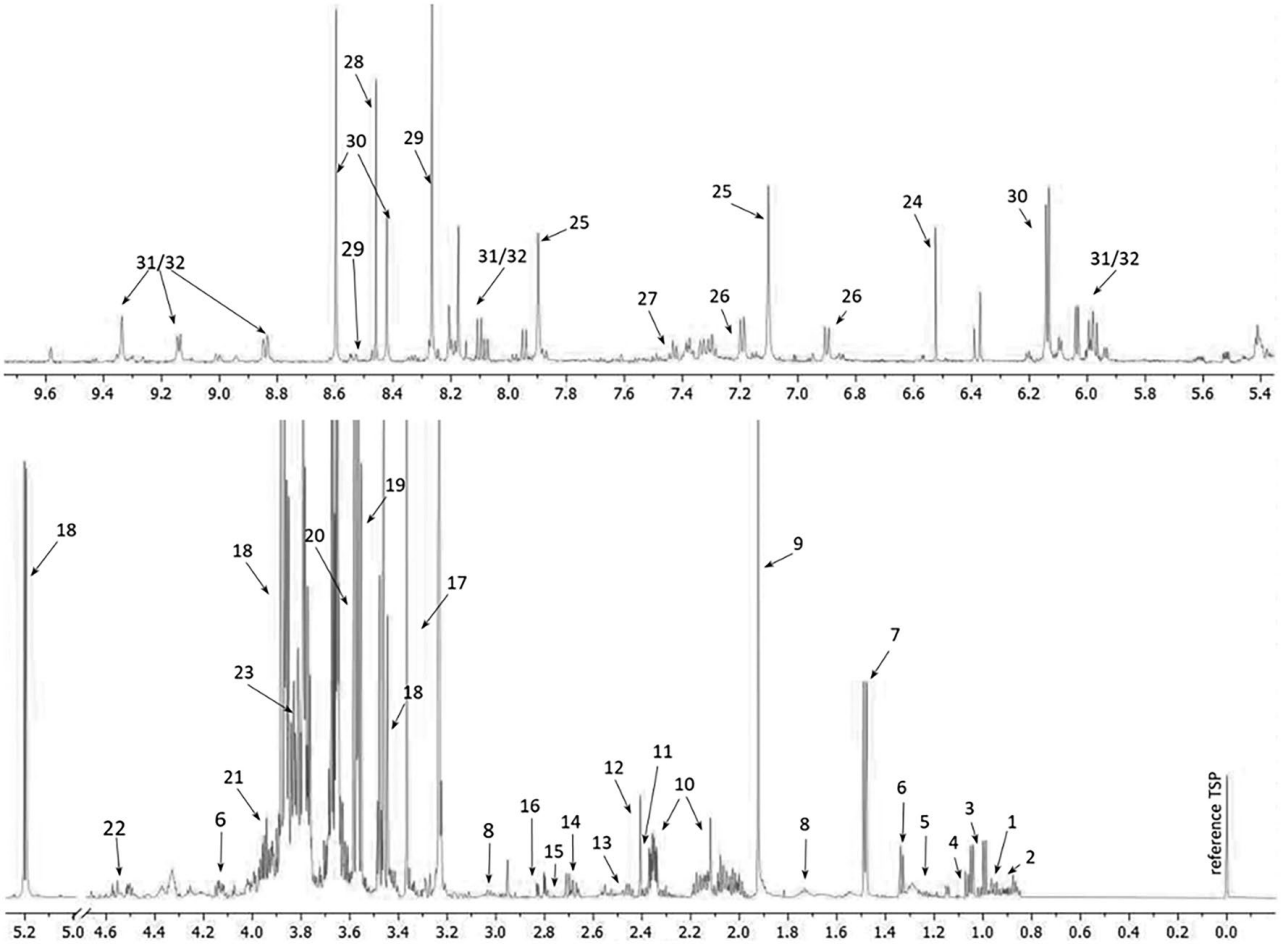
The representative 1D ^1^H NMR spectra of yeast strain.Arrows indicate signals assigned to the following metabolites: 1—Isoleucine; 2—Leucine; 3—Valine; 4—3-hydroxyisobutyrate; 5—Ethanol; 6—Lactate; 7—Alanine; 8—Lysine; 9—Acetate; 10—Glutamate; 11—Pyruvate; 12—Succinate; 13—Glutamine; 14—Aspartate; 15—Sarcosine; 16—Asparagine; 17—Methanol; 18—Trehalose; 19—Glycerol; 20—Glycine; 21—Serine; 22 —Proline; 23—Glucose; 24—Fumarate; 25—Histidine; 26—Tyrosine; 27—Phenylalanine; 28 —Formate; 29—ATP; 30—AMP; 31—NADP + ; 32—NAD + .

^1^H NMR analysis of intracellular metabolites enabled the identification of 31 metabolites. The following metabolites were identified: 15 amino acids (isoleucine, leucine, valine, alanine, lysine, glutamate, glutamine, aspartate, asparagine, glycine, serine, proline, histidine, tyrosine, and phenylalanine), 4 coenzymes (ATP, AMP, NADP^+^, NAD^+^), 4 metabolites from main metabolic pathways (glucose, pyruvate, succinate, fumarate), and 8 other metabolites (3-hydroxyisobutyrate, ethanol, lactate, acetate, sarcosine, trehalose, glycerol, formate). Methanol was also detected in the NMR analysis; however, since this compound is used as a solvent in the metabolite isolation protocol, we consider its presence as an artifact. The representative ^1^H NMR spectrum is presented in Fig. 2. Information about the chemical shift for each metabolite is available in the supplementary materials (Table S3).

**Figure 3:**
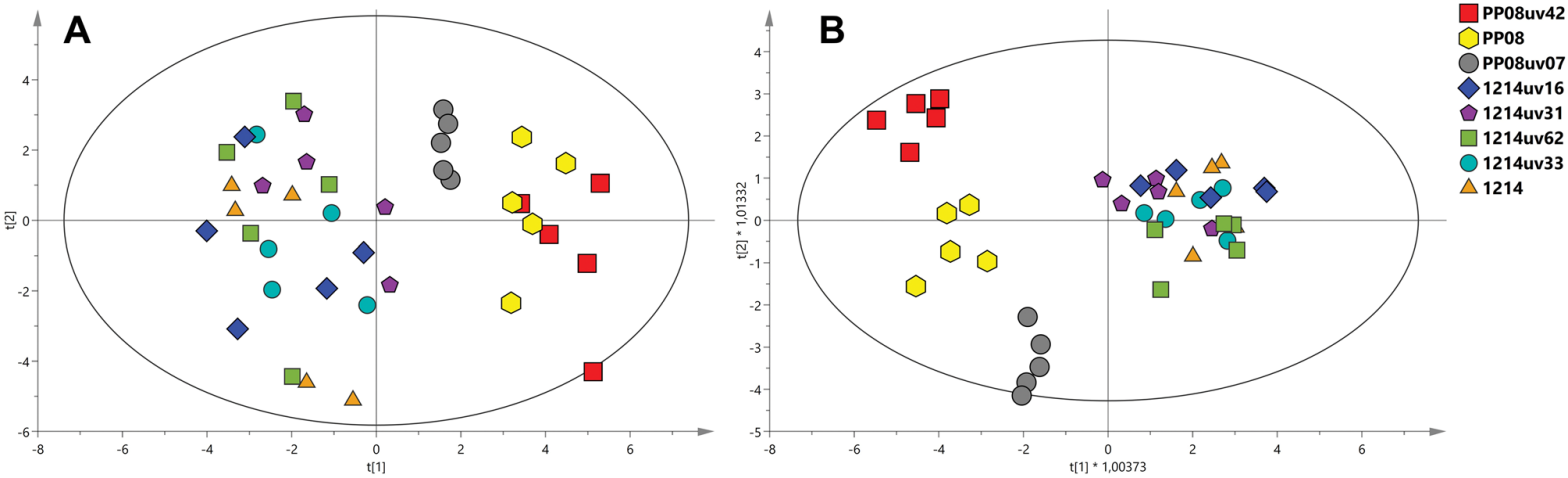
Score plots of ^1^H NMR metabolomic data from yest strains: (**A**) PCA (R^2^X = 0.415, Q^2^ = 0.235) and (**B**) OPLS-DA (R^2^X = 0.674, R^2^Y = 0.541, Q^2^ = 0.295 and CV-ANOVA p = 0.930.

The results of PCA and OPLS-DA analysis of ^1^H NMR metabolomic data from yeast strains are presented in Fig. 3. The first two principal components explain 25.6 and 15.9% of the observed variability in the PCA model. In both models strains derived from individual parental microorganism are grouped together. Moreover, strains derived from 1214 seem to form a more homogeneous group than strains derived from PP08.

**Figure 4:**
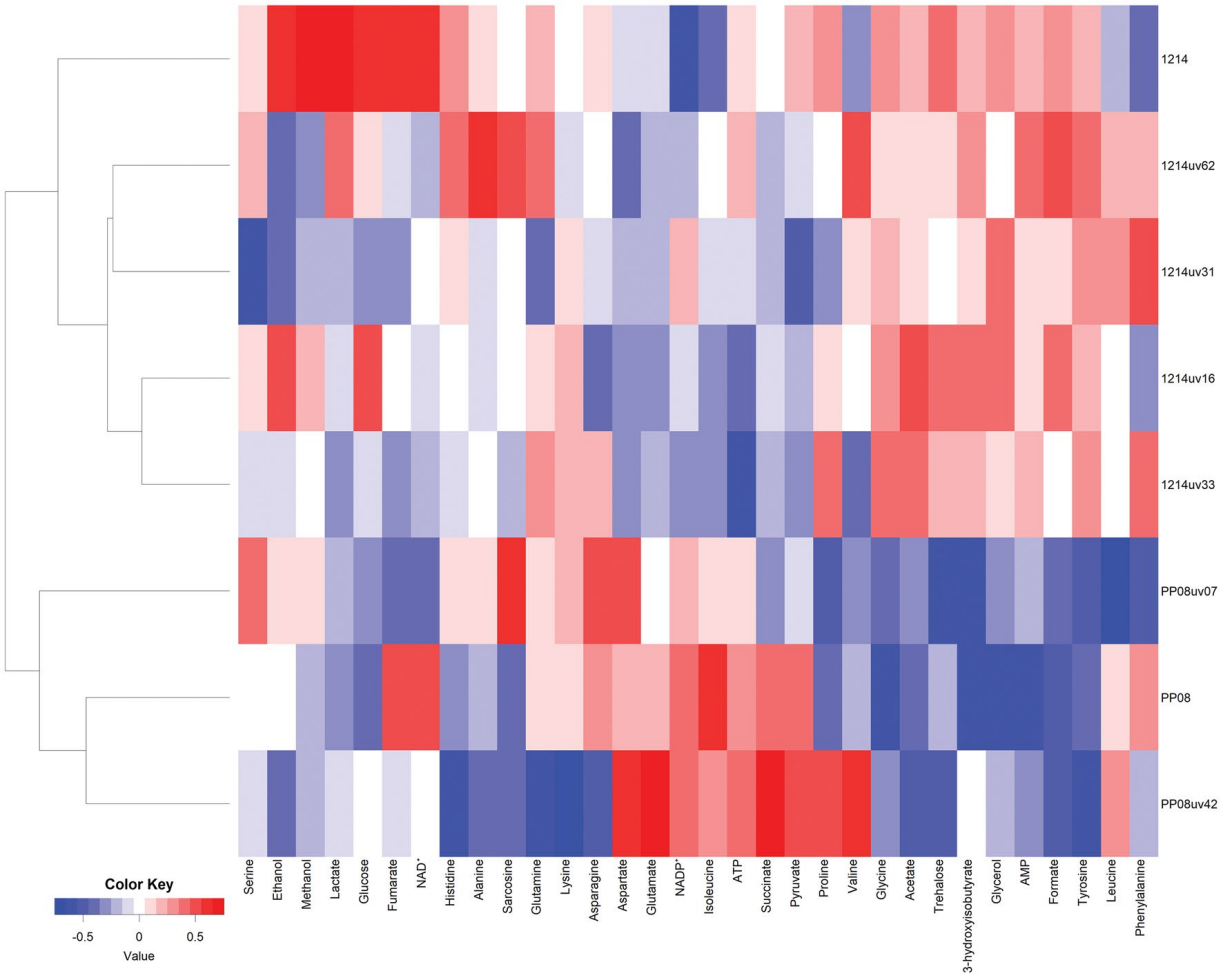
Heat map representing relative concentration of internal metabolites in different yeast strains.

The variability of intracellular metabolite concentrations observed for the analyzed set of strains is presented as a heatmap in Fig. 4. Hierarchical clustering of metabolomic data seems to confirm results obtained from PCA and OPLS-DA analysis; mutant strains are grouped with parental strain in one cluster.

Comparison of mutated yeast with parental strain revealed significant differences for particular metabolites. This analysis also showed that strains derived from PP08 show greater diversity than strains derived from 1214. In case of PP08 strain family significant differences were observed for 15 metabolites while for 1214 strain family significant differences was identified only for 7 metabolites. For detailed result see supplements materials: tables from S4 to S7.

Despite fermentation being an anaerobic process, metabolites required to close the TCA cycle, succinate and fumarate, were present in all samples. This observation confirms previous reports describing the presence of these two acids in yeast cells in anaerobic conditions^33^. The presence of fumarate and succinate may be a bit surprising since the TCA cycle is broken in anaerobic conditions (succinate dehydrogenase is not active). Although the synthesis of some amino acids (such as aspartate and glutamate) requires reactions included in the TCA cycle, the presence of succinate and fumarate seems unnecessary in this process. Yeast strains lacking enzymes required for fumarate production performed similarly to wild-type strains in anaerobic conditions^33^. It is interesting to explore the circumstances under which the presence of these metabolites in anaerobic conditions would be beneficial for the cell.

Another interesting metabolite identified in intracellular extracts with ^1^H NMR was 3-hydroxyisobutyrate. The concentration of this compound was relatively high in comparison to other metabolites since it could be detected using ^1^H NMR technique. 3-hydroxyisobutyrate was also detected by other research teams as an intracellular metabolite in yeast^34^. This compound is a part of the metabolic pathway of valine degradation leading to the formation of methylmalonate and succinate^35^. This mechanism is considered to be absent in *S. cerevisiae* since valine cannot be used as the sole source of carbon for yeast growth, nor all homologues of enzymes required in this pathway were identified in yeast. 3-hydroxyisobutyrate may be also derived from thymine, however the situation with thymine degradation pathway is the same as with valine, homologues of required enzymes in yeast were not identified. Results obtained for strain PP08uv42 may suggest that 3-hydroxyisobutyrate is derived from valine, since concentration of both metabolites was significantly increased (*p* < 2%) in comparison to parental strain PP08. To confirm this hypothesis experiments with isotopically labeled valine are required. If this is truly present in *S. cerevisiae* than the model of valine related pathways should be revised.

The fermentation industry continuously seeks innovative yeast strains to craft beverages offering unique sensory experiences. Highlighting microorganisms’ significance in green chemistry, our study underscores the enduring importance of random mutagenesis in developing yeast strains suitable for industrial applications. The results presented in this publication indicate that our current understanding of the mechanisms regulating VOCs production remains incomplete, mirroring gaps in the models of yeast metabolic pathways. Further analysis of strains 1214uv16, 1214uv33, and 1214uv31 could illuminate metabolic pathways influencing VOC production, potentially bridging existing knowledge gaps in yeast metabolism. This includes clarifying reactions leading to 2-ethylhexanol formation, understanding the regulation of phenylalanine-related VOCs production, and elucidating the synthesis of ethyl decanoate. Such information is crucial for refining metabolic models and lays a foundational stone for advancements in synthetic biology.

## Materials and methods

### Yeast strains

The list of yeast strains used in research is presented in Table 2.

**Table 2:** The list of yeast strains used.

No	Code	Species	Description
1	1214	*Saccharomyces cerevisiae*	Yeast strain used in ale production commercially available as Wyeast 1214 Belgian Abbey
2	PP08		Yeast strain isolated from natural environment around Wrocław (Poland) in 2017
3	1214uv16		Yeast strain obtained from 1214 by random UV induced mutagenesis
4	1214uv31		Yeast strain obtained from 1214 by random UV induced mutagenesis
5	1214uv33		Yeast strain obtained from 1214 by random UV induced mutagenesis
6	1214uv62		Yeast strain obtained from 1214 by random UV induced mutagenesis
7	PP08uv07		Yeast strain obtained from PP08 by random UV induced mutagenesis
8	PP08uv42		Yeast strain obtained from PP08 by random UV induced mutagenesis

### Yeast maintenance conditions

For long-term storage, yeast strains were preserved as cell suspensions with glycerol (15.75%) as a cryoprotectant and stored at −75 °C. For short-term storage, strains were transferred to a 3% malt extract solid medium, incubated at 28 °C for 48 h, and stored at 4 °C for no longer than 7 days.

### Yeast starter culture preparation

A single yeast colony was transferred from solid medium to a test tube containing 5 ml of YPG medium (yeast extract 1%, peptone 1%, glucose 2%) and incubated overnight at 28 °C without shaking. Then, 100 ml of 3% malt extract in a 300 ml Erlenmeyer flask was inoculated with the yeast suspension from the test tube. Yeasts were incubated at 28 °C for 24 h on an orbital shaker (160 rpm).

### Beer production in laboratory scale

Wort was prepared by dissolving food-grade powdered malt extract (Wytwórnia Ekstraktów Słodowych Sp. z o. o.) in tap water and boiling for 60 min. After boiling, the wort was diluted with boiled tap water to a final sugar concentration of 104 g/L (10° Bx). After cooling to 10 °C, 0.6 l of clean wort was transferred to sterile fermentation bottles, and a sterile ZnCl_2_ solution was added (to a final concentration of 0.27 mg/L). The wort was aerated with sterile air for 5 min. Then, the wort was inoculated with 50 ml of yeast suspension to reach a cell concentration of 10^7^ cells/ml. Fermentation bottles were sealed with an airlock and left for 7 days at room temperature (21 to 24 °C). The wort was manually agitated for 30 s every 24 h. After 7 days, the beer was transferred to 0.5 l storage bottles. 2.5 g of glucose was added per bottle for refermentation. Green beer was maturated for 7 days at room temperature and 21 days at 4 °C. Fermentation trials were prepared in three repeats.

### Biomass propagation for metabolite extraction

5 ml of liquid medium (3% malt extract) was transferred to test tube, inoculated with a single colony and incubated at 28 °C for 24 h without shaking. 50 ml of wort, prepared in the same way as for laboratory-scale beer production, was transferred to a 100 ml Erlenmeyer flask and inoculated with yest suspension from a single test tube. The yeast culture was incubated at room temperature (21–24 °C) for 24 h without shaking. After incubation, cell density was estimated by counting cells in Thoma hemocytometer. Cultures for metabolite isolation were prepared in five repeats.

### Internal metabolite extraction

Metabolite extraction was performed according to the protocol described by Smart and coworkers^36^ with modifications. Biomass was collected on a 0.45 µm cellulose acetate filter (Sartorius) using vacuum filtration. Cells were washed with 10 ml of ice-cold 0.9% NaCl solution, immediately transferred to a 5 ml polyethylene test tube, and suspended in 2.5 ml of a 50% methanol solution (−30 °C). The cell suspension was stored in an ice-salt bath and sonicated for 1 min (100 W, 20 kHz frequency, Microson Ultrasonic Cell Disruptor, model: XL2007, Misonix Inc.). Then, the suspension was centrifuged at 20,000 r.c.f., −4 °C, for 15 min (3–18 K, Cat. No.10290, Sigma-Laborzentrifugen). The clean solution was transferred to a fresh test tube, and the pellet was resuspended in 2.5 ml of a 50% methanol solution (−30 °C). Centrifugation was repeated, and the collected clean solution was combined with the first portion. Extracts were frozen at -80 °C and lyophilized. Powders were stored at −80 °C before analysis.

### Gas chromatography mass spectrometry (GC–MS)

The volatile compounds were adsorbed on a solid-phase microextraction fiber (SPME) according to Gasiński et al.^37^, using 30 µl of the internal standard (IS) (1 mg of 2-undecanone per 1 L of cyclohexane) and 2 ml of beer. The extraction of volatiles was performed for 20 min at a temperature of 40 °C. Volatile compounds were analyzed by gas chromatography-mass spectrometry using a GC-2010 Plus chromatograph coupled with a GCMS-QP2010 SE mass spectrometer (Shimadzu, Kyoto, Japan), equipped with a ZB-5 column (Phenomenex, Torrance, CA, USA) (30 m length × 0.25 mm inner diameter × 0.25 μm layer thickness). Helium was used as the carrier gas (1.78 cm^3^/min, initial pressure 100 kPa). The injection port was maintained at 195 °C. The volatiles were desorbed from the fiber (1 cm long DVB/CAR/PDMS fiber with 50/30 µm thickness of stationary phase; Supelco, Bellefonte, PA, USA) in the injection port for 2 min. The following oven temperature program for the GC analysis was used: 40 °C for 1 min, ramp up (8 °C/min) to 195 °C; hold (5 min). The ion source temperature was 250 °C, and the interface temperature was 195 °C. Scanning was performed in the range of 35–350 m/z using 70 mV electron ionization, with an event time of 0.3 s (scan rate of 1111).

### ^1^H NMR spectroscopy analysis of the yeast’s metabolites

To the evaporated extracts, 600 µL of PBS buffer (0.5 mol/L, 10% D2O, pH = 7.0, TSP = 0.3 mmol/L) were added and mixed for 1 min. In the next step, samples were centrifuged (10000 rpm, 4 °C, 5 min), and 550 µl of the upper phase was transferred into 5-mm NMR tubes (5SP, Armar Chemicals) for measurements. Until the measurements were taken, the samples were stored at 4 °C.

Standard ^1^H NMR experiments were performed on a Bruker AVANCE II 600.58 MHz spectrometer equipped with a 5 mm TBO probe at 298 K. All one-dimensional ^1^H NMR spectra were carried out using the cpmgpr1d (in Bruker notation) pulse sequence by suppression of water resonance by presaturation. Acquisition parameters were as follows: spectral width, 10 ppm; the number of scans, 128; acquisition time, 2.72 s per scan; relaxation delay, 3.5 s; and time-domain points, 64 K. The spectra were referenced to the TSP resonance at 0.0 ppm and manually corrected for phase and baseline (MestReNova v. 11.0.3).

### Data processing and multivariate statistical data analysis

All spectra from ^1^H NMR were exported to Matlab (Matlab v. 8.3.0.532) for preprocessing. Regions affected by solvent suppression were excluded (4.664–5.007 ppm), and alignment procedures involving the correlation of optimized warping (COW) and interval correlation shifting (icoshift) algorithms were applied^38,39^. The spectra consisted of 8,910 data points and were normalized using the probabilistic quotient method to overcome the issue of dilution^40^.

The multivariate and statistical data analysis were performed on a set of the 32 assigned metabolites. The concentration of the metabolite measured by NMR was obtained as the sum of the intensities of the non-overlapping resonances (or a part of partly overlapping resonances). The input for SIMCA-P software was a transformed data matrix (v 15.02, Umetrics, Umeå, Sweden).

After the separation of VOCs on GC, mass spectral analysis was used to identify volatile compounds. Identification was carried out by comparative analysis of retention indices with Kovats standards and NIST17 chemical standard libraries. Peak integration was performed using the Shimadzu Post Run Analysis software (Shimadzu, Kyoto, Japan). Statistical analysis of results, heat map, and bar chart were prepared with R software (4.3.2) and OriginPro (10.0.5.157) software. The heatmap was generated with the function ‘heatmap.2’ with default settings for hierarchical clustering. Distribution normality was checked with Shapiro test, equity of variance was analyzed with Brown-Forsythe test or Bartlett’s test. ANOVA was performed for data sets fitting test assumptions, otherwise Kruskal–Wallis test was used. For subgroup pairwise comparison Tukey’s HSD test (for data fitting ANOVA assumptions) or Wilcoxon test (for remaining data). Raw data regarding concentration of metabolites are supplied in tables S8, S9 in supplementary materials.

### Supplementary Information

Supplementary Table S1.

Supplementary TableS2.

Supplementary Table S3.

Supplementary Tables S4 and S5.

Supplementary Tables S6 and S7.

Supplementary Tables S8 and S9.

## Data Availability

All data generated or analyzed during this study are included in its supplementary information files.
