# Author Correction: Examination of internal metabolome and VOCs profile of brewery yeast and their mutants producing beer with improved aroma

**DOI:** 10.1038/s41598-024-67390-2

**Published:** 2024-07-17

**Authors:** Sławomir Jan Jabłoński, Karolina Anna Mielko-Niziałek, Przemysław Leszczyński, Alan Gasiński, Joanna Kawa-Rygielska, Piotr Młynarz, Marcin Łukaszewicz

**Affiliations:** 1 Department of Biotransformation, Faculty of Biotechnology, University of Wrocław, Wrocław, Poland; 2 Department of Biochemistry, Molecular Biology and Biotechnology, Faculty of Chemistry, Wrocław University of Science and Technology, Wrocław, Poland; 3 Department of Fermentation and Cereals Technology, Faculty of Biotechnology and Food Science, Wrocław University of Environmental and Life Sciences, Wrocław, Poland

Correction to: *Scientific Reports* 10.1038/s41598-024-64899-4, published online 25 June 2024

The Funding section in the original version of this Article was omitted. The Funding section now reads:

Funding

“Open access was financially supported by the fund of the University of Wrocław for scientific research and commercialization of its results.”

The original Article has been corrected.

